# Role of the Functional Toll-Like Receptor-9 Promoter Polymorphism (-1237T/C) in Increased Risk of End-Stage Renal Disease: A Case-Control Study

**DOI:** 10.1371/journal.pone.0058444

**Published:** 2013-03-05

**Authors:** Hsin-Yi Yang, Kuo-Cheng Lu, Herng-Sheng Lee, Shih-Ming Huang, Yuh-Feng Lin, Chia-Chao Wu, Donald M. Salter, Sui-Lung Su

**Affiliations:** 1 School of Public Health, National Defense Medical Center, Taipei, Taiwan, Republic of China; 2 Division of Nephrology, Department of Medicine, Cardinal Tien Hospital, School of Medicine, Fu Jen Catholic University, New Taipei City, Taiwan, Republic of China; 3 Department of Pathology, Tri-Service General Hospital, National Defense Medical Center, Taipei, Taiwan, Republic of China; 4 Department of Biochemistry, National Defense Medical Center, Taipei, Taiwan, Republic of China; 5 Division of Nephrology, Department of Medicine, Shuang Ho Hospital, Graduate Institute of Clinical Medicine, Taipei Medical University, New Taipei City, Taiwan, Republic of China; 6 Division of Nephrology, Department of Medicine, Tri-Service General Hospital, National Defense Medical Center, Taipei, Taiwan, Republic of China; 7 Center for Molecular Medicine, MRC IGMM, University of Edinburgh, Edinburgh, United Kingdom; The University of Texas MD Anderson Cancer Center, United States of America

## Abstract

Inflammation induced by infectious and noninfectious triggers in the kidney may lead to end stage renal disease (ESRD). Toll-like receptor 9 (TLR-9) a receptor for CpG DNA is involved in activation of immune cells in renal disease and may contribute to chronic inflammatory disease progression through an interleukin-6 (IL-6) dependent pathway. Previous studies indicate that -1237T/C confers regulatory effects on TLR-9 transcription. To date the effect of TLR-9 polymorphisms on ESRD remains unknown. We performed a case-control study and genotyped 630 ESRD patients and 415 controls for -1237T/C, -1486T/C and 1635G/A by real-time PCR assays and assessed plasma concentration of IL-6 by ELISA. Haplotype association analysis was performed using the Haploview package. A luciferase reporter assay and real-time PCR were used to test the function of the -1237T/C promoter polymorphism. A significant association between -1237T/C in TLR-9 and ESRD was identified. The TCA, TTA and CCA haplotype of TLR-9 were associated with ESRD. ESRD patients carrying -1237TC had a higher mean plasma IL-6 level when compared with -1237TT. The TLR-9 transcriptional activity of the variant -1237CC allele is higher than the -1237TT allele. The results indicate that in a Han Chinese population the presence of the C allele of -1237T/C in the TLR-9 gene increases susceptibility towards development of ESRD. In vitro studies demonstrate that -1237T/C may be involved in the development of ESRD through transcriptional modulation of TLR-9.

## Introduction

In Taiwan chronic kidney disease (CKD) is a major public health problem due to its high prevalence, high rates of healthcare utilization, high risk of progression to end-stage renal disease (ESRD) and poor prognosis [1]. The rising tide of CKD not only adds burden to global health-care resources but also has major impact on patients and their families. CKD is classified as a multifactorial disease as a combination of genetic and environmental factors influence the onset and development of ESRD [2], [3]. It is now recognized that inflammation may be established before the onset of renal disease and could be a causal factor in the development of CKD. Sensors of the innate immune system, including Toll-like receptors (TLRs), provide danger recognition platforms on immune and renal cells. These can integrate and translate the diverse triggers of renal inflammation by regulating cell activation and production of proinflammatory cytokines and chemokines [4]–[6].

Mammalian TLRs comprise a large family of at least 11 members. Members of the TLR family play an important role in both innate and adaptive immune responses. Their genes have been found to be polymorphic [7]. TLRs recognize a wide variety of pathogen associated molecular patterns (PAMPs) from bacteria, viruses and fungi as well as some host molecules. TLR-9, expressed within the endosomal compartment, recognizes unmethylated CpG motifs present in bacterial DNA and intracellular viral antigens [8]. Recent studies have suggested roles for TLR-9 in the development of renal diseases such as glomerulonephritis [9] and lupus nephritis [10]. Single nucleotide polymorphisms (SNPs) in TLR genes affect the susceptibility to and severity of inflammatory diseases by influencing the function of these receptors. The profile of currently known genetic polymorphisms in TLR-9 has been proposed to associate with severe clinical phenotypes [11], [12] and TLR-9 polymorphisms appear to affect IgA nephropathy progression [13].

In a human embryonic kidney cell line (HEK293) model system the ability to respond to physiological and therapeutic TLR-9 ligands depends on TLR-9 SNPs [14]. -1237T/C confers regulatory effects on TLR-9 transcription [15]. Indeed the C allele of the -1237T/C polymorphism generates several regulatory sites, including an IL-6-responsive element [16] and was associated with chronic renal disease in a limited candidate gene study [17]. The affect of TLR-9 polymorphisms on ESRD however remains unknown. Therefore we investigated the predictive value of TLR-9 gene polymorphisms on ESRD in a Han Chinese population and undertook in vitro experiments to study potential mechanisms of any associations.

## Methods

### Study Subjects

This case-control study included 630 ESRD patients (325 females and 305 males; age 64.62±14.51 years) recruited from the Cardinal Tien Hospital and five hemodialysis centers in Taipei, Taiwan. CKD was defined according to KDOQI (Kidney Disease Outcomes Quality Initiative) definitions and estimated glomerular filtration rate (eGFR) was calculated using the Modification of Diet in Renal Disease (MDRD) Study equation [18]. ESRD was defined as eGFR <15 ml/min/1.73 m^2^ associated with clinical signs of uremic syndrome requiring dialysis. The enrolled patients were stable (without clinical complications), aged over 20 and had been on hemodialysis (HD) for more than 6 months. Patients with autoimmune disease, malignancy and acute or chronic infection were excluded. The causes of ESRD were diabetes mellitus in 244 patients (38.7%), chronic glomerulonephritis in 199 patients (31.6%), hypertensive nephropathy in 76 patients (12.1%), systemic nephropathy in 51 patients (8.1%) and other and unknown causes in 60 patients (9.5%). The 415 healthy control subjects (217 females and 198 males; age 74.91±7.50 years) with no history of renal disease and whose eGFR was ≥60 ml/min/1.73 m^2^ were recruited from the Center of Physical Examination at Cardinal Tien Hospital. The healthy control subjects showed no microalbuminuria, proteinuria or hematuria and had normal abdominal/renal ultrasonography. 24.5% healthy controls reported a history for hypertension and 13.3% for diabetes mellitus.

### Ethics Statement

The study was reviewed and approved by the institutional ethical committee of Cardinal Tien Hospital (CTH-98-3-5-045). After full explanation of the study written informed consent was obtained from all participants. All clinical and biological samples were collected and DNA was genotyped following patient consent.

### SNP Genotyping

Genomic DNA was extracted from the peripheral blood of patients and controls using the QIAamp DNA Blood Mini Kit (QIAGEN Inc., Hilden, Germany) according to the manufacturer's instructions. Genotyping for the TLR-9 -1237T/C (rs5743836), -1486T/C (rs187084) and -1635G/A (rs352140) was performed by real-time polymerase chain reaction (PCR). Genotypes were determined using the LightCycler 1.2 System (Roche Diagnostics, Salt Lake City, UT, USA). Primer and detection probes for each polymorphism were based on Hamann et al. and Soriano-Sarabia et al. [19], [20]. Melting curve analyses for TLR genes were performed using 2.5 mM of each detection probe. After an initial denaturation at 95°C for 10 minutes at a ramp rate of 4.4°C/s, temperature was dropped to 45°C at a ramp rate of 1°C/s and finally led to 80°C with one acquisition per degree Celsius. Genotyping was done by laboratory personnel blinded to case status and a random 10% of the samples were repeated to validate genotyping procedures.

### Peripheral blood mononuclear cell Culture and Plasma Interleukin-6 Concentration

Peripheral blood mononuclear cells (PBMCs) were prepared from venous blood by Ficoll-Hypaque density-gradient centrifugation (Amersham Pharmacia Biotech, Little Chalfont, UK). PBMCs were plated at a density of 1×10^6^ cells/ml in 12-well cell culture plates with RPMI 1640 supplemented with 10% heat-inactivated fetal calf serum (FCS) and 100 µg/ml streptomycin. Plasma concentration of interleukin-6 (IL-6) was determined by ELISA using human Quantikine ELISA kit (R&D Systems, Minneapolis, MN, USA).

### Expression Analysis by Real-time Quantitative Reverse Transcription Polymerase Chain Reaction

1×10^6^ PBMCs cultured as above were serum starved and treated with 100 pg/ml IL-6 for 24 hours. Total RNA was isolated using TriZOL reagent (Invitrogen Cor., Carlsbad, CA, USA). Two microgram of total RNA was reverse transcribed by use of the High Capacity cDNA Reverse Transcription Kit (Applied Biosystems, Foster City, CA, USA) into cDNA. Real-time PCR was performed with the Maxima® SYBR green qPCR master mix (Fermentas, Glen Burnie, MD, USA), using an ABI 7500 real-time PCR system (Applied Biosystems). Primer sequences were as follows: TLR-9 forward 5′-CCCGCTACTGGTGCTATCC-3′ and reverse 5′-CCTTCCTCTTTCCACTCCC-3′; β-actin forward 5′-AGTTGCGTTACACCCTTTCTTG-3′ and reverse 5′-TCACCTTCACCGTTCCAGTTT-3′. Thermocycling was performed at 95°C for 10 min, 40 cycles of 95°C for 15 s and 60°C for 60 s to measure the fluorescence signal. The dissociation stages, melting curves and quantitative analyses of the data were performed using 7500 system software v1.2.3 (Applied Biosystems). Expression of β-actin was used as internal control. TLR-9 gene expression normalized by β-actin was calculated by using the 2^−ΔΔCt^ method.

### Transient Transfection and Luciferase Assay

Two TLR-9 promoter reporters (from 52260706 to 52260840, 135 bp), TLR-9-1237TT-LUC and TLR-9-1237CC-LUC, were amplified by PCR from human genomic DNA including the SNP of interest (-1237T/C) and subcloned into a pGL3 basal reporter cut at *Xho*I and *Hind*III sites. The 5′ and 3′ primers used for PCR were: 5′- CCgCTCgAgATGGGAGCAGAGACATAATGGA-3′ and 5′-CCCAAgCTTCTGCTTGCAGTTGACTGTGT-3′. HEK293 cells were grown in Dulbecco's modified Eagle's medium (DMEM) supplemented with 10% charcoal/dextran-treated fetal bovine serum. The cells in each well (24-well plate) were transfected with jetPEI (PolyPlus-transfection, Illkirch, France) according to the manufacturer's protocol; total DNA was adjusted to 1.0 µg by addition of the pGL3 reporter. 24 hours post transfection HEK293 cells were treated with IL-6 (5 ng/ml) for an additional 18 hours. Luciferase activity was assessed using the Promega Luciferase Assay kit and expressed as mean relative light units (RLU) of two transfected sets. Results shown are representative of at least three independent experiments.

### Statistical Analysis

Statistical analysis was performed with SPSS for windows version 18.0 (SPSS, Chicago, IL, USA). Demographic, clinical data and plasma IL-6 concentration between groups were compared by Student's t test or Mann-Whitney U test. The results for continuous variables are given as means ± SD. The genotype distributions were tested for Hardy-Weinberg equilibrium. The comparison of the allele and genotype frequencies between the different groups was evaluated by Chi-square test or Fisher's exact test when appropriate. The odds ratios (ORs) and corresponding 95% confidence intervals (CIs) for assessing the effect of the genotype distribution and allele frequencies on ESRD were calculated by logistic regression analysis with adjustment for relevant significant variables. Statistical significance was defined at the 95% level (P<0.05). Linkage disequilibrium (LD) and haplotype analyses were performed using Haploview software [21] (http://www.broad.mit.edu/mpg/haploview/) and WHAP (http://pngu.mgh.harvard.edu/~purcell/whap/), respectively. Associations of TLR-9 promoter polymorphism with TLR-9 mRNA expression were assessed by the Kruskal-Wallis (K-W) test.

## Results

### Demographic Characteristics

The characteristics of the 1,045 subjects are presented in Table 1. There was no significant difference in gender and diastolic blood pressure between the two groups. Significant differences in age, BMI, smoking history, systolic blood pressure, fasting blood sugar, eGFR, BUN, serum creatinine, total cholesterol and triglycerides were observed between ESRD patients and controls (P<0.001).

**Table 1:** Clinical and biochemical parameters in ESRD patients and control subjects.

	Patients n = 630	Controls n = 415	P value
Male (%)	48.4	47.6	0.80
Age (yrs)	64.62±14.51	74.91±7.50	<0.001
Body mass index (kg/m^2^)	21.71±5.39	24.18±3.11	<0.001
Current or former smoker (%)	22.8	14.0	<0.001
Systolic blood pressure (mmHg)	140.86±33.25	129.14±15.64	<0.001
Diastolic blood pressure (mmHg)	75.39±10.84	75.14±11.96	0.72
Fasting plasma glucose (mg/dL)	143.76±56.94	102.56±22.59	<0.001
eGFR	7.81±8.33	84.67±16.90	<0.001
BUN (mg/dL)	65.53±19.10	16.27±6.22	<0.001
Serum creatinine (mg/dL)	9.40±2.60	0.86±0.32	<0.001
Serum total cholesterol (mg/dL)	166.49±33.38	187.44±32.98	<0.001
Serum triglycerides (mg/dL)	152.33±95.92	116.50±58.55	<0.001

Quantitative data are mean ± SD.

### Association Analyses of TLR-9 Gene Polymorphisms with Susceptibility to ESRD

The distributions of TLR-9 -1237T/C, -1486T/C and 1635G/A genotypes and allele frequencies were compared between ESRD patients and controls (Table 2). The genotypic distributions of the gene polymorphisms in the patients and controls fit the Hardy-Weinberg equilibrium. The genotype and allelic distributions of -1237T/C in TLR-9 was significantly different between the patients and healthy controls (adjusted OR = 4.49, 95% CI = 1.75–11.49, P = 0.002 and adjusted OR = 4.36, 95% CI = 1.72–11.08, P = 0.002, respectively). There were no significant differences of genotypic and allelic frequencies in either the TLR-9 -1486T/C or 1635G/A between ESRD patients and controls. The dominant genetic models showed that -1486T/C may be a protective factor for ESRD (adjusted OR = 0.71, 95% CI = 0.53–0.97, P = 0.03).

**Table 2:** Genotype distributions and allele frequencies for the TLR-9 gene in ESRD patients and control subjects.

Genotypes	Patients n or (%)	Controls n or (%)	Crude OR (95% CI)	P value	*Adjusted OR (95% CI)	P value
**T-1237C**				<0.001		
TT	591	408	1		1	
TC	39	7	3.85 (1.70–8.68)	0.001	4.49 (1.75–11.49)	0.002
Alleles						
T-allele	97%	99%	1		1	
C-allele	3%	1%	3.76 (1.67–8.44)	0.001	4.36 (1.72–11.08)	0.002
**T-1486C**				0.10		
TT	276	155	1		1	
TC	290	208	0.78 (0.60–1.02)	0.07	0.72 (0.52–1.00)	0.05
CC	64	52	0.69 (0.46–1.05)	0.08	0.68 (0.41–1.13)	0.14
Alleles						
T-allele	67%	62%	1		1	
C-allele	33%	38%	0.82 (0.69–0.99)	0.04	0.80 (0.64–1.00)	0.05
#D model	276/354	155/260	0.77 (0.59–0.99)	0.04	0.71 (0.53–0.97)	0.03
&R model	566/64	363/52	0.79 (0.54–1.17)	0.23	0.81 (0.51–1.30)	0.39
**G1635A**				0.90		
GG	245	158	1		1	
GA	306	206	0.96 (0.73–1.25)	0.75	0.94 (0.68–1.30)	0.72
AA	79	51	1.00 (0.67–1.50)	0.99	0.97 (0.59–1.58)	0.90
Alleles						
G-allele	63%	63%	1		1	
A-allele	37%	37%	0.99 (0.82–1.19)	0.90	0.97 (0.78–1.21)	0.81
#D model	245/385	158/257	0.97 (0.75–1.25)	0.79	0.95 (0.70–1.29)	0.73
&R model	551/79	364/51	1.02 (0.70–1.49)	0.90	1.00 (0.63–1.58)	0.99

* Data are expressed as n or (%) and have been adjusted by gender, age, BMI, and smoking status.

# D model = Dominant model.

& R model = Recessive model.

Although a higher eGFR was observed for patients with the TLR-9 -1237TT genotype this did not reach statistical significance and overall there was no significant association between TLR-9 -1237T/C and the degree of renal function in the ESRD group (results not shown). Stratifying patients by the underlying cause of their renal disease demonstrated marginal associations with genotype and both glomerulonephritis (*p* = 0.04) and hypertension (P = 0.03) but not with diabetic (P = 0.08) or systemic nephropathy (P = 0.13) (results not shown).

### Haplotype Analysis of TLR-9

The haplotype analysis of TLR-9 polymorphisms in ESRD patients and control subjects is shown in Table 3. The frequency of haplotype “TCA” was 27.8% in the ESRD patients compared to 34.6% in the controls (OR = 0.73, 95% CI = 0.60–0.88, P = 0.001). In contrast haplotypes TTA and CCA were more common in ESRD patients (6.5% and 2.5%, respectively) than in controls (1.9% and 0.6%, respectively) (OR = 3.47 and 4.45, 95% CI = 2.02–5.97 and 1.70–11.67, P<0.001 and <0.001, respectively). Other haplotypes showed no significant difference (Table 3).

**Table 3:** Haplotype frequencies in TLR-9 of ESRD patients and control subjects.

Haplotype			Frequencies			
T-1237C	T-1486C	G1635A	Patients	Controls	P value	OR (95% CI)
T	T	G	0.603	0.603	0.90	1.00 (0.83–1.19)
T	C	A	0.278	0.346	0.001	0.73 (0.60–0.88)
T	T	A	0.065	0.019	<0.001	3.47 (2.02–5.97)
T	C	G	0.023	0.023	0.90	1.02 (0.57–1.83)
C	C	A	0.025	0.006	<0.001	4.45 (1.70–11.67)

### Association of TLR-9 -1237 T/C Polymorphism with Plasma IL-6 Level

**Figure 1:**
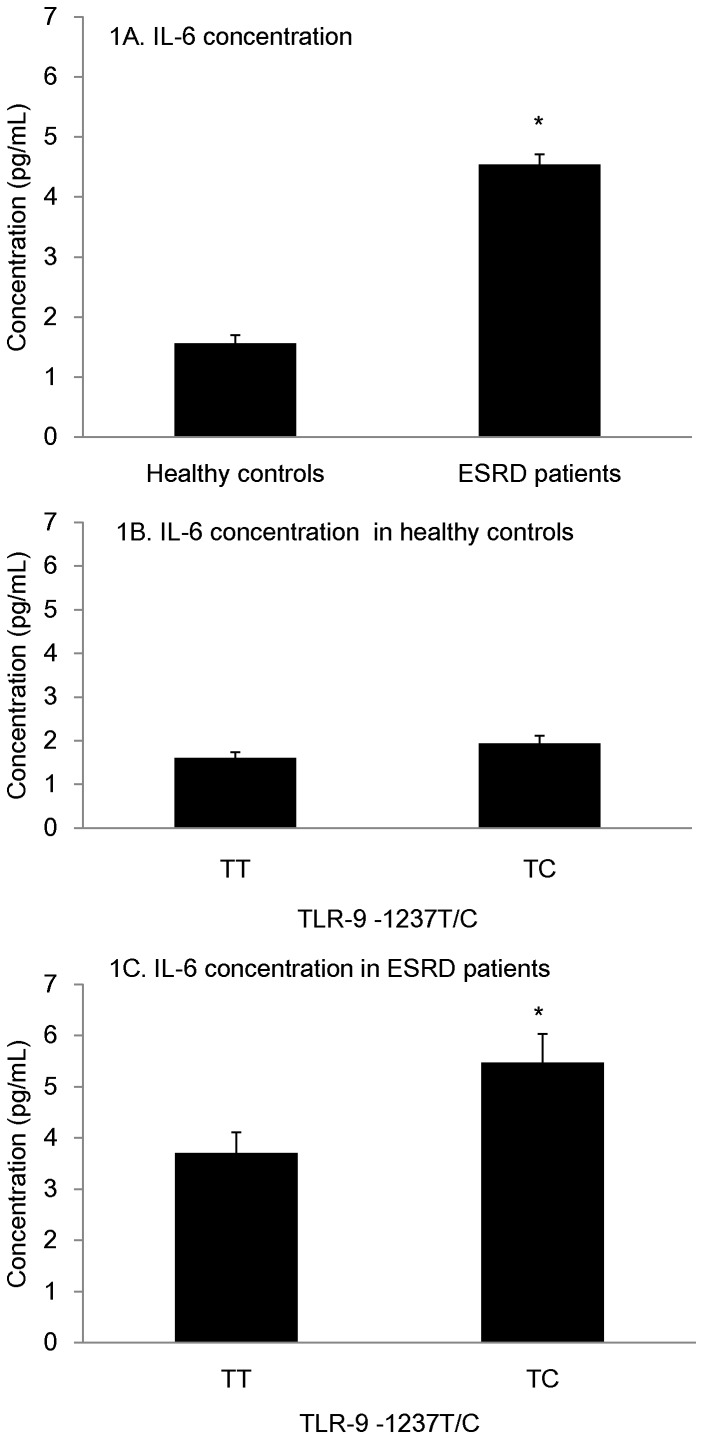
Effects of TLR-9 -1237T/C genotype on the level of plasma IL-6 in healthy controls and ESRD patients. IL-6 concentration was quantified by ELISA. Results are expressed as mean ± SEM. The columns represent the mean value and the lines represent standard error of mean. A: Compared to the healthy controls, the ESRD patients showed a significant increase in plasma IL-6 level (P<0.001). B: The difference of mean level of IL-6 between TT (n = 22) and TC (n = 7) carriers was not significant (P = 0.87) in healthy controls. C: The difference of mean level of IL-6 between TT (n = 18) and TC (n = 14) carriers showed significant difference (P = 0.01) in ESRD patients. *P<0.05.

We evaluated whether the TLR-9 -1237 T/C polymorphism might be associated with IL-6 levels in the plasma of ESRD patients. Data are shown in figure 1. Compared to healthy controls (1.56±0.24 pg/ml) the ESRD patients (4.54±0.38 pg/ml) showed a significantly higher level of plasma IL-6 (P<0.001, figure 1A). The plasma IL-6 level did not differ significantly in healthy controls between different TLR-9 -1237T/C genotypes (TT: 1.55±0.10 pg/ml; TC: 1.60±0.11 pg/ml, P = 0.87, figure 1B). The ESRD patients carrying -1237TC had a higher mean plasma IL-6 level (5.47±0.56 pg/ml) when compared with -1237TT (3.71±0.40 pg/ml; P = 0.01, figure 1C). No significant association was shown for other TLR-9 polymorphisms with plasma IL-6 concentration (results not shown).

### TLR-9 mRNA Expression in PBMCs

**Figure 2:**
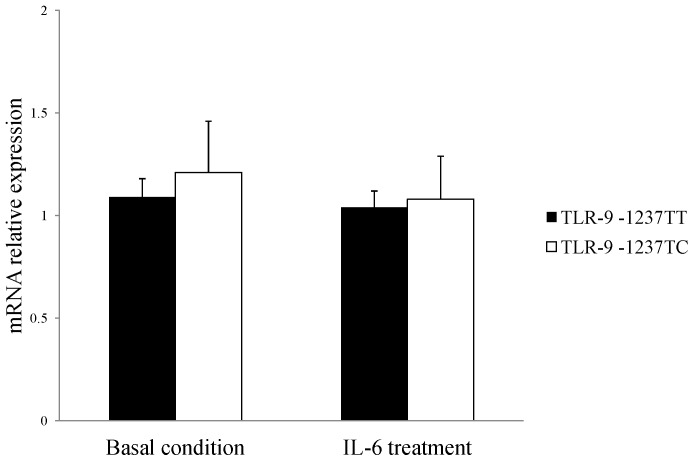
Comparison of PBMC TLR-9 mRNA expression between different genotypes of -1237T/C. β-actin gene expression was used as an internal control gene. TLR-9 mRNA expression in TT or TC PBMCs in the absence or presence of IL-6 (100 pg/ml) treatment was assessed. Values shown are mean ± SEM. Experiments were performed in triplicate.

The results of TLR-9 gene expression among 32 ESRD patients are shown in figure 2. Under basal conditions and following IL-6 treatment there was no significant difference in the expression of TLR-9 mRNA between the TT (n = 18) and TC (n = 14) genotypes (basal condition, TT = 1.09±0.09, TC = 1.21±0.25; IL-6 treatment, TT = 1.04±0.08, TC = 1.08±0.21. mean ± SEM relative mRNA expression). Similar results were seen in samples from healthy controls (TT = 8, TC = 7) (results not shown). Under basal conditions or following IL-6 treatment there was no significant difference in the expression of TLR-9 mRNA between the genotypes (mean ± SEM relative mRNA expression: basal condition, TT = 1.12±0.15, TC = 1.19±0.18; IL-6 treatment: TT = 1.08±0.19, TC = 1.16±0.21).

### Comparison of Promoter Activity of TT and CC Alleles of the TLR-9 Promoter -1237T/C

**Figure 3:**
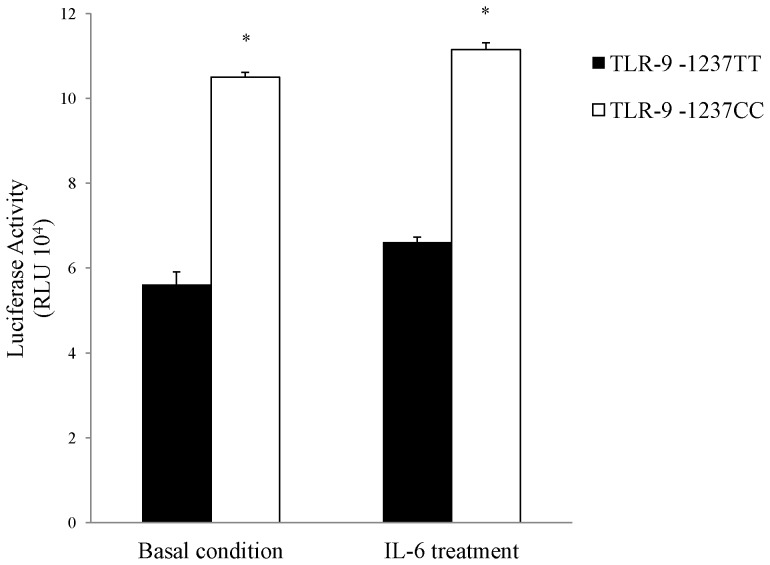
Effects of the -1237T/C genotype in TLR-9 promoter on luciferase activity in cultured HEK293 cells. Luciferase reporters containing a TLR-9 promoter sequence with the wild-type T allele or risk C allele at SNP -1237T/C were transfected into HEK293 cells. The mean ± SEM is given for each construct from three experiments. *P<0.05.

To establish whether the TLR-9 SNPs were functionally important we investigated whether IL-6 influenced TLR-9 promoter activity using a luciferase reporter assay in HEK293 cells. The results are shown in figure 3. The luciferase activity of the C allele was significantly higher in the absence (10.50±1.06) and presence of IL-6 (11.15±0.16) in comparison to that of the T allele (basal condition 5.61±0.29 and following IL-6 treatment 6.61±0.12) (P = 0.01). However there was no significant difference in TLR-9 promoter activity between -1237TT and -1237TC following IL-6 treatment (P = 0. 10 and 0.49, respectively).

## Discussion

Based on a previous study by our group [17] we have examined possible associations between three TLR-9 SNPs and ESRD in a Han Chinese population. Furthermore, in view of the known interactions between TLR-9 and IL-6 we also studied the association between TLR-9 -1237T/C and plasma IL-6 levels in ESRD and performed functional studies using this SNP. Our study has shown a statistically significant association between a polymorphism in the promoter of TLR-9 gene (-1237T/C) and ESRD. A weaker association between TLR-9 -1486T/C and ESRD was seen in dominant genetic models. The study groups were matched for sex but there were significant differences in BMI, smoking status and age between ESRD patients and healthy controls. Indeed it is recognized that patients with ESRD are often malnourished [22], [23] and malnourishment is associated with poor survival in these patients [24]. We have used a non-matched case-control design and applied multiple logistic regression to adjust for confounders. Interestingly a study analyzing the quality of life of irritable bowel syndrome patients compared results when groups were matched and non-matched and found identical results. Indeed the matching procedure slightly diminished the statistical power of the results [25]. In the current study the frequency of TLR9 -1237T/C was 1%. The allele frequency of this SNP in the Han Chinese population (0.007–0.03) [17], [26], [27] is significantly different from Caucasians (0.10–0.15) and African Americans (0.25–0.39) [28], [29]. This indicates the existence of a geographic/ethnic-specific difference in TLR-9 genotypes that may in part reflect ethnic diversity to ESRD susceptibility or other conditions where TLR-9 pathways may be involved.

There is emerging evidence that TLR-9 may play an important role in renal disease and a number of inflammatory conditions of other organ systems. Activation of TLR-9 induces progression of renal disease in MRL-Fas (lpr) mice [30]. TLR-9 has been shown to be involved in antigen-induced immune complex glomerulonephritis and lupus nephritis through regulation of both humoral and cellular immune responses [31]. With respect to TLR-9 SNPs, the -1237T/C polymorphism has been shown to be associated with asthma [29], Crohn's disease [32] and HIV infection [33]. The TLR-9 2848 AA genotype is associated with significantly higher expression of TLR-9 and the frequency of intracellular IgM positive B cells in patients with Primary Biliary Cirrhosis [34] whilst the TLR-9 1635G/A polymorphism appears to play a role in the susceptibility to Systemic lupus erythematosus (SLE) in a Chinese population [35]. Less is known regarding TLR-9 SNPs and renal disease. We have previously identified an association between CKD and the -1237T/C of TLR-9 [17] whilst another group has shown that +1174A/G is associated with increased risk for progression of IgA nephropathy in Japanese patients [36] and patients with a TT genotype at 1635G/A show more severe renal damage and poorer therapeutic outcomes [37].

The haplotype of three polymorphisms in TLR-9 was associated with ESRD in the current study. Our data indicate that ESRD patients carrying the TCA haplotype had a lower risk of ESRD than those not carrying this haplotype whilst patients carrying the TTA or CCA haplotype had a higher risk of ESRD than those not carrying these haplotypes. Holla et al. (2009) demonstrated previously that the TLR-9 TTA haplotype may increase susceptibility to chronic periodontitis whereas the TLR-9 TCG haplotype has a protective effect against this condition [38]. Specific haplotypes in the TLR-9 gene might affect host defense mechanisms and influence susceptibility or resistance to infections. Common TLR-9 alleles with a frequency higher than 5% however do not appear to contribute significantly to the genetic risk involved in susceptibility to SLE or lupus nephritis [39]. Further work is required to fully understand the role of TLR-9 haplotypes on the genetic susceptibility to ESRD.

TLR-9 has been mapped to chromosome 3p21.3. It spans approximately 5 kb and has two exons, the second of which is the major coding region. To date several transcription factors such as AP-1, Sp-1, NF-κB, GATA and CRE are recognized to regulate expression of the TLR-9 gene [40]. In the current study we assessed the functionality of the TLR-9 -1237T/C polymorphism for effects on transcriptional activity. Our data suggest that transcriptional activity of the variant C allele is higher than that of the wild-type T allele. This may be a consequence of creation of an NF-κB binding site with the C allele [41]. Indeed higher transcriptional activity was seen in the presence of the CC allelic variant. These results are consistent with observations of a moderate increase in promoter activity associated with the -1237CC genotype of TLR-9 in asthma patients [15]. Higher promoter activity of the TT allelic variant at -1237T/C in atopic eczema [42] may relate to different pathogenic mechanisms and indicate the need for further investigation in this area. In silico analysis of the human TLR-9 promoter reveals that the C allele of the -1237T/C polymorphism generates several new regulatory sites including an IL-6-responsive element [16]. In the study of Carvalho et al (2011) PBMCs containing the TC allele but not those with the TT allele increased TLR-9 gene expression in response to IL-6 [16]. In contrast no association between -1237T/C genotype and TLR-9 mRNA level or promoter activity following IL-6 treatment was identified in the current study. The reason for this discrepancy is not clear but differences in cell sources, detection methods, and time course of experiments in addition to the complexity of gene regulation in vivo may be relevant.

IL-6 is a pleiotropic cytokine that plays a central role in modulating inflammatory responses. Several studies have indicated that IL-6 is a reliable predictor of mortality in ESRD [43], [44]. B-cell activation with a TLR-9 agonist significantly increases production of IL-6 in ESRD patients when compared to normal age-matched controls [45]. Associations between genetic polymorphisms and plasma IL-6 levels have been identified [46]. SNPs in the IL-6 gene are associated with plasma IL-6 in myocardial infarction survivors [47] and IL-6 plasma levels are modulated by a polymorphism in the NF-κB1 gene [48]. Our data revealing that ESRD patients carrying -1237TC in TLR-9 gene had a higher mean plasma IL-6 level than those carrying -1237TT are consistent with previous observations [16]. SNPs in the promoter region affecting TLR-9 transcriptional activity might explain inter-individual variability in the production of IL-6 in ESRD patients. However whether a TLR-9/IL-6 signal amplification loop such as that which regulates B cell proliferation [16] is active in patients with renal disease remains to be investigated.

In conclusion, we found the -1237T/C SNP of the TLR-9 gene is significantly associated with ESRD in a Han-Chinese population and that -1237T/C may be involved in the development of ESRD through transcriptional modulation of TLR-9. These observations provide new insights into the role of TLR-9 polymorphisms in renal disease that have the potential to provide new avenues for treatment and may also allow identification of individuals at risk.
